# Coronary artery disease, genetic risk and the metabolome in young individuals

**DOI:** 10.12688/wellcomeopenres.14788.2

**Published:** 2019-02-01

**Authors:** Thomas Battram, Luke Hoskins, David A. Hughes, Johannes Kettunen, Susan M. Ring, George Davey Smith, Nicholas J. Timpson

**Affiliations:** 1 MRC Integrative Epidemiology Unit, University of Bristol, Bristol, UK; 2 Population Health Sciences, Bristol Medical School, University of Bristol, Bristol, UK; 3 Center for Life Course Health Research, Faculty of Medicine, University of Oulu, Oulu, Finland; 4 Biocenter Oulu, University of Oulu, Oulu, Finland

**Keywords:** Coronary artery disease, metabolomics, genetics, childhood and adolescence, ALSPAC

## Abstract

**Background: **Genome-wide association studies have identified genetic variants associated with coronary artery disease (CAD) in adults – the leading cause of death worldwide. It often occurs later in life, but variants may impact CAD-relevant phenotypes early and throughout the life-course. Cohorts with longitudinal and genetic data on thousands of individuals are letting us explore the antecedents of this adult disease.

**Methods: **148 metabolites, with a focus on the lipidome, measured using nuclear magnetic resonance ( ^1^H-NMR) spectroscopy, and genotype data were available from 5,907 individuals at ages 7, 15, and 17 years from the Avon Longitudinal Study of Parents and Children (ALSPAC) cohort. Linear regression was used to assess the association between the metabolites and an adult-derived genetic risk score (GRS) of CAD comprising 146 variants. Individual variant-metabolite associations were also examined.

**Results: **The CAD-GRS associated with 118 of 148 metabolites (false discovery rate [FDR] < 0.05), the strongest associations being with low-density lipoprotein (LDL) and atherogenic non-LDL subgroups. Nine of 146 variants in the GRS associated with one or more metabolites (FDR < 0.05). Seven of these are within lipid loci: rs11591147 *PCSK9, *rs12149545 *HERPUD1-CETP, *rs17091891 *LPL, *rs515135 *APOB, *rs602633 *CELSR2-PSRC1, *rs651821 *APOA5, *rs7412 *APOE-APOC1. *All associated with metabolites in the LDL or atherogenic non-LDL subgroups or both including aggregate cholesterol measures. The other two variants identified were rs112635299 *SERPINA1 *and rs2519093 *ABO. *

**Conclusions: **Genetic variants that influence CAD risk in adults are associated with large perturbations in metabolite levels in individuals as young as seven. The variants identified are mostly within lipid-related loci and the metabolites they associated with are primarily linked to lipoproteins. Along with further research, this knowledge could allow for preventative measures, such as increased monitoring of at-risk individuals and perhaps treatment earlier in life, to be taken years before any symptoms of the disease arise.

## Introduction

Coronary artery disease (CAD) is the leading cause of adult death worldwide and is a gross contributor to global morbidity ^1^. Many of the risk factors have long been established to be modifiable exposures such as low-density lipoprotein (LDL) cholesterol levels, smoking and hypertension ^2^. In the developed world, the average age of developing Angina Pectoris, often the first clinical sign of CAD, is typically over 60 ^3^. However, there is evidence that “fatty streaks”, the precursors to atherosclerosis and thus CAD, form in almost all adolescents from developed countries ^4^. Furthermore, there is evidence that the development of atherosclerotic plaques in coronary arteries is prolonged over the life course ^5^. Thus, it is unsurprising that risk factors for CAD, including obesity and serum low-density lipoprotein levels, have been associated with an increased rate of plaque formation in children ^6^. Also, a recent study suggested that higher BMI early in life was causally associated with adverse cardiovascular health ^7^. These observations strongly suggest that at least some CAD risk factors may be contributing to disease development within children and there is potential for early life intervention, even if it involves nothing other than heightened clinical surveillance (not screening) by measured genetic burden.

Genome-wide association studies (GWAS) have been conducted to explore common forms of heritable contributions to this complex disease ^8, 9^. Over 100 genetic variants have been identified as being reliably associated with an increased risk of CAD in adults ^8, 9^. These variants, which are likely to be exerting their influence through a diverse collection of mechanisms, are common and exert relatively small effects on disease outcome singularly, but together these variants explain over 10% of CAD heritability ^8, 9^.

It is unclear what effect these variants are having on CAD-relevant phenotypes at an earlier age (i.e. latent disease) or the longitudinal nature of the associations. Elsewhere, work analysing variation near the *FTO* locus and BMI has shown that risk alleles don’t always have fixed effects on outcomes throughout life ^10^. This may also be the case for other traits, like CAD. This has clinical importance because at risk individuals may gain from treatment or monitoring at various time-points across their life course. There are also implications for applied epidemiology using genetics. Currently, it is often assumed the effect of a genetic variant is fixed across the life course, but whilst the nature of the code itself may be static, the penetrance may be variable, one possible source of this variation comes from gene-environment interactions.

Proton nuclear magnetic resonance ( ^1^H-NMR) spectroscopy offers a cost effective, high throughput technology to analyse multiple metabolic measures from a single sample, providing quantitative information on 149 metabolites ^11– 13^. The platform focuses largely on lipoproteins and fatty acids and provides the opportunity to examine individual components of lipoproteins in addition to aggregate measures. With such detailed measures of both genotypes and phenotypes, studies have already begun to successfully associate genotypic and metabolic profiles to disease phenotype, such as type 2 diabetes ^14^.

Furthermore, single nucleotide polymorphisms (SNPs) have been used as instrumental variables (in a technique called Mendelian randomisation ^15^) to begin to appraise the causal relationship between metabolites and CAD in adults ^16^. This technique, along with new methods to quantify metabolites are starting to build evidence for the causal associations between metabolites and CAD that are beyond the well-known LDL-C and CAD relationship.

There is a clear need to explore the nature of established adult genetic associations at earlier ages. Thus, this study set out to use a detailed collection of genetic and metabolomic data to assess how genetic risk of CAD is associated with established and potential risk factors for CAD in young individuals (aged 7, 15, 17).

## Methods

### Study sample

The study used a single cohort: the Avon Longitudinal Study of Parents and Children (ALSPAC). ALSPAC recruited pregnant women in the Bristol and Avon area, United Kingdom, with an expected delivery date between April 1991 and December 1992. Over 14,000 pregnancies have been followed up (both children and parents) throughout the life-course. Full details of the cohort has been published previously ^17^. This study focuses on the children of these pregnancies. EDTA plasma samples were collected for metabolite extraction at ages 7, 15 and 17. Individuals at ages 15 and 17 were fasted prior to sample collection, but individuals at age 7 were not. Samples were aliquoted at 200μl or 500μl and stored below -70°C. Of the 7,176 participants available, 1,269 were removed due to incomplete data, leaving 5,907 for the analysis. Data at the three ages were combined in order to maximise the power of the study (N = 5,907). This was achieved by taking an individual’s metabolite data at the earliest time point possible. Full details of their characteristics are in Table 1.

**Table 1.** Cohort characteristics.

	f7	tf3	tf4	P
N	4685	858	364	
mean age (sd)	7.54 (0.33)	15.48 (0.36)	17.86 (0.41)	
N female (%)	2265 (48.3)	461 (53.8)	200 (54.9)	
mean CAD score (sd)	0.36 (0.39)	0.37 (0.39)	0.35 (0.40)	0.535

Genotype and metabolite data were available from individuals that attended 3 clinics at different ages. N = sample size and is naturally smaller by age as the largest sample (youngest age) was used as a core collection to which non-overlapping participants from later clinics with ^1^H-NMR data were added. f7, tf3 and tf4 are all clinics where individuals aged 7, 15 and 17 respectively were invited in to have various measurements taken. CAD (coronary artery disease) score refers to a genetic risk score comprised of 146 coronary artery disease associated genetic variants weighted by their association with the disease. The P value represents a group-wise comparison between the different CAD score values of the clinics.

Ethical approval for the study was obtained from the ALSPAC Ethics and Law Committee and from the UK National Health Service Local Research Ethics Committees. Full references of committee approval can be found on the ALSPAC website. Written informed consent was obtained from both the parent/guardian and, after the age of 16, children provided written assent. Please note that the study website contains details of all the data that is available through a fully searchable data dictionary.

### Genotyping

Children were genotyped using the Illumina HumanHap550 quad genome-wide SNP genotyping platform (Illumina Inc., San Diego, CA, USA) by the Wellcome Trust Sanger Institute (WTSI; Cambridge, UK) and the Laboratory Corporation of America (LCA, Burlington, NC, USA). Participants were excluded due to having at least one of: incorrectly recorded sex, minimal or excessive heterozygosity, disproportionate levels of individual missingness (>3%), evidence of cryptic relatedness or non-European ancestry. SNPs with a minor allele frequency (MAF) <1%, a genotype missingness >1% and a call rate <95% were removed and only SNPs that passed an exact test of Hardy-Weinberg equilibrium (P<5x10-7) were included.

For imputation, genotypes of ALSPAC mothers and children were combined. Haplotypes were estimated using ShapeIT (v2.r644), which utilises relatedness during phasing. A phased version of the 1000 genomes reference panel (Phase 1, Version 3) was obtained from the Impute2 reference data repository. Imputation was performed using Impute V2.2.2 against the reference panel (all polymorphic SNPs excluding singletons), using all 2186 reference haplotypes (including non-Europeans).

### Genetic risk scores

A GWAS meta-analysis conducted using data from UK biobank and CARDIoGRAMplusC4D identified 148 variants associated with CAD at genome-wide significance (P < 5×10 ^-8^) ^9^. 146 of these variants were present in the genotype data after quality control (see above) and were included in the genetic risk score. The effect size of each variant in relation to CAD was used to weight the variants – specifically the natural log of the odds ratio (OR) was used. These weightings were multiplied by the variant dosage and a CAD-GRS was produced for each individual by summing all the weighted variant values. All the loci are outlined in Supplementary Table 1.

### Metabolite measures

NMR analyses of the metabolic measures was carried out at the University of Eastern Finland quantifying 149 metabolites from serum samples of the participants. The process has been described elsewhere ^12^. Briefly, the samples are prepared automatically with a Gilson Liquid Handler 215, whereby 300μl of sodium phosphate NMR buffer are mixed with 300μl of serum sample. Once prepared the samples are inserted into the SampleJet™ (Bruker BioSpin GmbH, Germany) sample changer. Finally, the data are measured using a Bruker AVANCE III spectrometer. Metabolite data contains known risk factors for CAD, such as LDL-cholesterol, but also many other metabolites, as well as multiple lipoprotein subclasses. Due to the unreliability of the signal, pyruvate was removed from the analyses, leaving 148 metabolites. All abbreviations of metabolites used can be found in Supplementary Table 2.

### Lipoprotein groupings

To examine the association between the GRS and different classes of lipoproteins, lipoproteins were split into six groups based on their size and density. The groups are labelled LDL, atherogenic non-LDL, large very low-density lipoproteins (VLDL), small high-density lipoproteins (HDL), large HDL, and very large HDL ( Supplementary Table 3). Groups were split in this way as it is hypothesised that: 1. The roles of lipoproteins of different sizes and densities differ 2. Only certain lipoprotein particles (here LDL and atherogenic non-LDL particles) cross into the intima, or inner most layer of a blood vessel ^18, 19^, which is required for atherosclerosis.

### *HMGCR* variant analysis

We sought to gauge whether a lipid lowering therapy may impact the metabolome similarly in young individuals and adults, and thus potentially reduce risk of CAD in later life. Two additional variants, external to the GRS, within the *HMGCR* locus, rs17238484 and rs12916, were chosen as proxies for statin use, as has been done previously ^20^. As these were separate from the GRS, the variants were not weighted by their association with CAD and their impact on metabolite concentrations was assessed separately to all the other variants.

### Statistical analyses

Metabolites were rank normalised prior to analyses to approximate normal distributions and to remove the impact of outliers. Linear regression models were used to estimate the association between metabolites in adolescence and genotype. Separately, metabolite concentrations were fitted against the CAD-GRS and each of the individual variants. Age was the only covariate in the models. An FDR-corrected P value < 0.05 was calculated using the Benjamini and Hochberg method ^21^.

The metabolites measured here do not necessarily represent independent phenotypes, as many are the product of the same biological event or pathway. As such, to estimate the number of independent metabolites or features present in our dataset we performed a hierarchical clustering and tree cutting analysis on the metabolite abundance data, in R ^22^. Specifically, distances among metabolites was estimated by 1. subtracting the absolute Pearson's correlation coefficient from one, 2. performing hierarchal clustering on a matrix of those distances with the hclust() function and the method "complete", 3. followed by a tree cutting step at the height of 0.2 with the function cutree(). The functions hclust() and cutree() are both available in the 'stats' package ^22^.

All analyses were conducted in R ^22^ (version 3.2.2).

## Results

### Biological and phenotypic grouping of metabolites

5,907 individuals aged 7, 15 and 17 had NMR-measured metabolite data and genotype data ( Table 1). Many metabolites share similar metabolic pathways, thus we attempted to deduce the number of independent features. Using hierarchical clustering we observe 41 independent metabolite clusters (-0.2 < r < 0.2), 22 of which are made up of a single metabolite.

When grouping lipoproteins based on their size and density we found a large overlap between the biological groupings and the clusters, with the lipoproteins within each group mostly mapping to a single cluster. This is with the exception of atherogenic non-LDL particles, where the metabolites overlap largely with clusters containing LDL and VLDL particles. Supplementary Table 3 shows the number of independent metabolite clusters represented by each grouping.

### CAD-GRS metabolite associations

**Figure 1.**
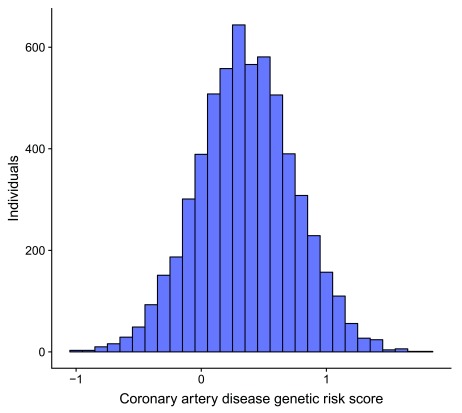
The distribution of the coronary artery disease genetic risk score amongst the individuals (N = 5,907) in the study. The score was made from 146 common genetic variants that associated with coronary artery disease in adults. Each variant was weighted by the effect size of its association with the disease.

A GRS produced from 146 CAD-associated variants, distribution shown in Figure 1, associated with 118 of the 148 metabolites tested (FDR < 0.05) ( Figure 1). The 118 metabolites were observed in 20 independent metabolite clusters (-0.2 < r < 0.2), seven of which contained single metabolites. The majority of the associated metabolites are either lipoproteins or fatty acids; the only two not in these categories were isoleucine and glycoprotein acetyls. The full table of results can be found in Supplementary Table 4.

**Figure 2.**
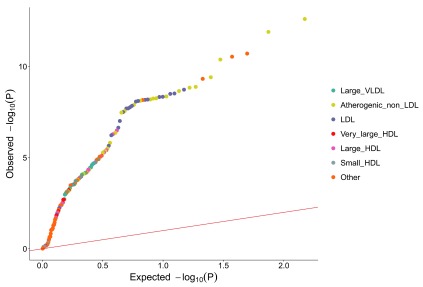
Association between 148 metabolites and a coronary artery disease genetic risk score. QQ-plot where each dot represents an association between one of the 148 metabolites and the genetic risk score comprised of 146 common variants. 98 of the 148 metabolites were lipoproteins and put into six groups based on size and density ( Supplementary Table 3). The “other” group contains the rest of the 50 metabolites. LDL = low-density lipoprotein, VLDL = very low-density lipoprotein, HDL = high-density lipoprotein, Other = non-lipoprotein metabolites.

When considering our lipoprotein biological groupings, it was observed that the GRS associated most strongly with atherogenic non-LDL particles and LDL ( Figure 2). Furthermore, there was good evidence that the median effect size on the groups differed (Kruskal-Wallis test, P = 4.8 × 10 ^-14^) and the median effect size on LDL and atherogenic non-LDL are larger than those observed for the other four groups (post-hoc Dunn’s test, FDR < 0.05), but are not different to each other (P = 0.86).

### Individual variant-metabolite analysis

**Figure 3.**
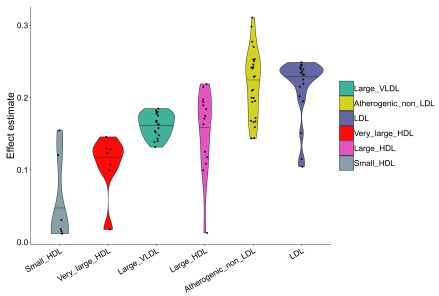
The association between 98 lipoprotein measures split into six subgroups and a coronary artery disease genetic risk score. The lipoproteins were organised into six groups based on size and density ( Supplementary Table 3). LDL = low-density lipoprotein, VLDL = very low-density lipoprotein, HDL = high-density lipoprotein.

To explore the variants driving the association between the GRS and various metabolites, all of the metabolites were regressed against each variant individually ( Figure 3). In total there was good evidence that nine variants associated with at least one metabolite (FDR < 0.05). Seven of these are within lipid loci: rs11591147 *PCSK9,* rs12149545 *HERPUD1-CETP,* rs17091891 *LPL,* rs515135 *APOB,* rs602633 *CELSR2-PSRC1,* rs651821 *APOA5,* rs7412 *APOE-APOC1.* All associated with metabolites in the LDL or atherogenic non-LDL subgroups or both, including aggregate cholesterol measures LDL-C, VLDL-C and IDL-C. rs2519093 *ABO* associated with three VLDL cholesterol measures and rs112635299 *SERPINA1* associated with glycoprotein acetyl and phenylalanine concentrations. Full tables of results for these nine variants found in Supplementary Table 5– Supplementary Table 13.

**Figure 4.**
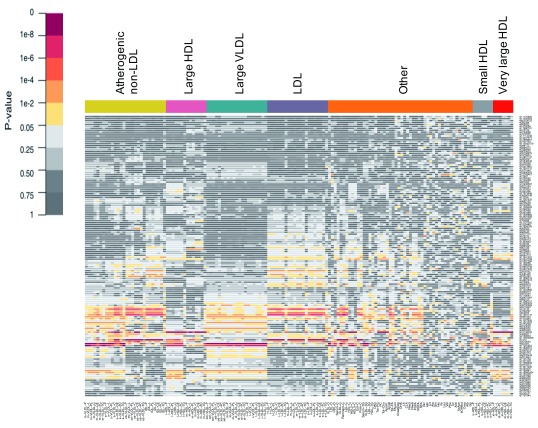
The association between all SNPs and all metabolites. Each metabolite was regressed against each SNP and the P values from these analyses are presented here. Grey indicates a high P value and red a low one. The lipoproteins were grouped as previously ( Supplementary Table 3).

### Potential for intervention

To assess the potential impact of early life intervention using agents that target lipoproteins, the association between rs17238484 and rs12916 *HMGCR* and the 148 metabolites was investigated. Neither of the SNPs associated with any metabolites at FDR < 0.05. At P < 0.05, rs17238484 and rs12916 associated with 17 and 42 metabolites respectively. Mostly, the presence of the effect allele (G and T respectively) associated with a decrease in metabolite levels within the lipoprotein subclasses LDL and atherogenic non-LDL particles. ( Supplementary Table 14, Supplementary Table 15). Supplementary Figure 1 shows the association between these variants and all metabolites alongside the other nine variants associated with one or more metabolites.

To assess whether the NMR measurements were representative of what is routinely measured in the clinic, a comparison between clinically measured LDL (a composite of NMR measures) and NMR LDL measures (23 measures) in individuals aged around seven were made. The NMR measures explained 80% of the variance of the composite LDL measure. The effect estimates from the association between all the SNPs and the NMR measures explained 93% of the variance of the effect estimates for the clinically measured LDL and all the SNPs.

### Age sensitivity analyses

In these analyses, we combined data at ages 7, 15 and 17. There were 4,685, 858 and 364 individuals from each age group respectively. To understand if grouping the individuals in this way impacted results we conducted sensitivity analyses using only individuals from each age group. We observed no strong evidence for a difference between the median metabolite levels at different age groups (Kruskal-Wallis test, P = 0.823), and there was minimal evidence for a difference between the association of the CAD-GRS and metabolites between age groups (Kruskal-Wallis test, P = 0.051). The extent of these differences for each metabolite is displayed in Supplementary Figure 2. The effect estimates for associations between the GRS and lipoprotein groups was largely consistent between age groups ( Supplementary Figure 3).

## Discussion

In this study a GRS of CAD, made from 146 variants identified in a previous GWAS ^9^, associated with 118 metabolites in a sample of 5,907 individuals aged 7, 15 and 17. These metabolites were mostly lipoproteins, with stronger associations occurring in LDL and atherogenic non-LDL particles subtypes. Nine of the variants were associated with one or more metabolites. When these variants were removed from a CAD-GRS, the association between the residual GRS and the metabolites attenuated to the null, strongly suggesting these nine variants were driving CAD-related metabolomic differences in young individuals.

The association between circulating metabolite levels and CAD has been demonstrated many times, especially with lipoproteins ^2, 23, 24^. Therefore, it is potentially unsurprising that Figure 1 suggests that all metabolites measured were associated with CAD variants, especially as the NMR platform contains a greater proportion of lipoproteins and lipids than anything else. However, to see such a perturbation in metabolite profiles in young individuals (aged 7, 15 and 17) suggests that there are long term effects of metabolites on CAD risk and thus early-life intervention of abnormal metabolite levels could be useful in preventing or delaying onset of this highly heritable disease. Of the nine metabolites that associated most strongly with the GRS, none of them were part of the LDL subgroup, however six were part of the atherogenic non-LDL subgroup previously hypothesised to be dangerous ( Supplementary Table 4). Although further analysis on the relevance of many of these metabolites to CAD needs to be done before drawing any strong conclusions.

The accumulation of lipoprotein particles, particularly LDL, within the intima has long been observed in atherosclerotic plaques ^23^. *In vivo* experiments suggest that not all lipoprotein particles can cross the intima ^18, 19^. Interestingly, the CAD-GRS associated most strongly with LDL and atherogenic non-LDL particles, both of which are hypothesised to be small enough to cross the intima. Furthermore, there is good evidence from randomised controlled trials and Mendelian randomization studies that lowering LDL-C (a conglomerate of all the cholesterol found within all sizes of LDL and some atherogenic non-LDL particles) reduces risk of CAD ^16, 25^. Thus, our results suggest genetic variants associated with CAD can drive an increase in metabolites that have evidence for causally influencing the disease.

Only 9 of the 146 variants associated with CAD, showed good evidence they associated with NMR measured metabolites in young individuals in this study. A recent GWAS of metabolites that featured 112 of our 149 metabolites was conducted in adults (mean age = 44.6) ^26^. All nine genetic variants identified in our study had good evidence for association with the same or similar metabolites in the adult GWAS. Interestingly, the GWAS identified five additional variants that were present in our study but had little evidence for association with metabolites. As only five more genetic variants were identified, it suggests many variants associated with CAD are acting through pathways independent of the metabolites measured here. In total, in the adult GWAS, the five SNPs associated with 89 metabolites. Of these 89 associations the direction of effect was the same for all but one within children and the 95% confidence intervals overlapped for 80 of the associations. Therefore, the discrepancy between the studies seems to be primarily due to power differences (the GWAS conducted in ~15,000 adults). The other discrepancies could be due to chance differences, or the effect of CAD-associated genetic variants on metabolites may vary temporally. Thus, more work is required to elucidate if it could be preferential to target some pathways within critical windows of time.

We assessed whether statin use might have a similar effect in young individuals as in adults in reducing LDL-C levels, to explore whether early-life drug-intervention may be a possibility for some individuals. A previous study by Swerdlow *et al.* showed that alleles rs17238484-G and rs12916-T ( *HMGCR*) associate with a decrease in LDL-C ^20^. Here we observed weak evidence that these variants associate with LDL and intermediate-density lipoprotein (IDL) subtypes in young individuals. Along with the association between the *PCSK9* variant and metabolites, it suggests that treatments attempting to target metabolites to reduce risk of CAD or prevent other adverse CAD-related outcomes, may have similar influences within young individuals, even if the effect is reduced. These results agree with the current treatment of familial hypercholesterolemia, whereby statins are administered at young ages ^27^. Unfortunately, there may be negative side effects of administering statins to younger individuals, with evidence linking statins to increases in risk of both type 2 diabetes and myopathy. Nevertheless, the consequences, negative and positive, of administering these agents early in life to “seemingly healthy” individuals need to be examined. There is the hypothetical potential that administering treatment early in life could delay onset of disease for at risk individuals.

Even though it is unlikely clinicians will prescribe pharmaceutical agents for CAD to very young people, the variants identified in this study could be used to select those who would benefit from a less dangerous lipoprotein lowering treatment. If no treatments became available, the identification of high-risk individuals could still be used to monitor them so that intervention could begin before symptoms start to arise. Furthermore, notification of those at risk could increase caution amongst parents and individuals over environmental exposures such as diet, physical activity and smoking.

## Limitations

The study combined metabolite data from young people aged 7, 15 and 17. Even though age was used as a covariate in the main models, sensitivity analysis revealed a potential difference in CAD-GRS associations with metabolites at different ages.

These data also combine metabolite data that was collected after fasting and non-fasting. There is evidence that fasting and non-fasting metabolite data are similar ^28^, but the study should be replicated using only fasting or only non-fasting data.

Rank-normalisation of the metabolite data removes the influence of outliers on the data but prevents true quantification of association between genotype and metabolite concentrations, i.e. with the addition of one risk allele the level of metabolite X increases by Y.

There is redundancy in the metabolite data, as many of the metabolites are highly correlated. This leads to an increase in false negatives when correcting for multiple testing. To reduce this, the Benjamini and Hochberg (FDR) method ^21^ was used to correct for multiple tests, rather than a more stringent family-wise error rate correction method such as Bonferroni correction. Furthermore, the study investigated how the GRS of CAD influenced lipoproteins grouped based on previous biological knowledge.

## Conclusion

A CAD-GRS associated with differential abundance of 118 metabolites in young individuals. The majority of these metabolites were lipoproteins and fatty acids, and it associated most strongly with lipoproteins that are hypothesised to causally influence CAD development. We believe these results warrant further research into whether identification of high-risk individuals, identified by their genetic profile, can benefit from increased monitoring and early life intervention, either by pharmaceutical agents or by behavioural changes.

## Data availability

ALSPAC data access is through a system of managed open access. The steps below highlight how to apply for access to the data included in this data note and all other ALSPAC data. The datasets presented in this article are linked to ALSPAC project number B2714, please quote this project number during your application. The ALSPAC variable codes highlighted in the dataset descriptions can be used to specify required variables.

1. Please read the ALSPAC access policy (PDF, 627kB) which describes the process of accessing the data and samples in detail, and outlines the costs associated with doing so.
2. You may also find it useful to browse our fully searchable research proposals database, which lists all research projects that have been approved since April 2011.
3. Please submit your research proposal for consideration by the ALSPAC Executive Committee. You will receive a response within 10 working days to advise you whether your proposal has been approved.

If you have any questions about accessing data, please email alspac-data@bristol.ac.uk.

The ALSPAC data management plan describes in detail the policy regarding data sharing, which is through a system of managed open access.

All code for the analysis is freely available on GitHub: https://github.com/thomasbattram/CAD_analysis

Archived code at time of publication: http://doi.org/10.5281/zenodo.1410263 ^29^

Licence: MIT

## Consent

Written informed consent was obtained from both the parent/guardian and, after the age of 16, children provided written assent. Children were invited to give assent where appropriate. Study members have the right to withdraw their consent for elements of the study or from the study entirely at any time. Full details of the ALSPAC consent procedures are available of the study website.

## Supplementary material

**Supplementary File 1: File containing the following supplementary figures –**

**Supplementary Figure 1.** SNPs associated with one or more metabolites at false discovery rate (FDR) < 0.05, along with the 2 SNPs within the *HMGCR* region (rs12916, rs17238484).

**Supplementary Figure 2.** A forest plot comparing the effect estimates for the association between the coronary artery disease (CAD) genetic risk score and 149 metabolites within each age.

**Supplementary Figure 3.** Comparison of effect estimates (transformed so all estimates are positive) for the association between the coronary artery disease-genetic risk score (CAD-GRS) and the lipoprotein sub-groups stratified by age.

**Supplementary File 2: File containing the following supplementary tables –**

**Supplementary Table 1.** Coronary artery disease (CAD)-associated loci

**Supplementary Table 2.** Nuclear magnetic resonance (NMR) measured metabolites

**Supplementary Table 3.** Biological grouping of lipoprotein subclasses

**Supplementary Table 4.** The effect estimates and P values for the associations between the coronary artery genetic risk score and 149 metabolites.

**Supplementary Table 5**. All associations between rs112645299 *SERPINA1* and metabolites (false discovery rate (FDR) < 0.05)

**Supplementary Table 6**. All associations between rs11591147 *PCSK9* and metabolites (false discovery rate (FDR) < 0.05)

**Supplementary Table 7**. All associations between rs12149545 *HERPUD1-CETP* and metabolites (false discovery rate (FDR) < 0.05)

**Supplementary Table 8**. All associations between rs17091891 *LPL* and metabolites (false discovery rate (FDR) < 0.05)

**Supplementary Table 9**. All associations between rs515135 *APOB* and metabolites (false discovery rate (FDR) < 0.05)

**Supplementary Table 10**. All associations between rs2519093 *ABO* and metabolites (false discovery rate (FDR) < 0.05)

**Supplementary Table 11**. All associations between rs602633 *CELSR2-PSRC1* and metabolites (false discovery rate (FDR) < 0.05)

**Supplementary Table 12**. All associations between rs651821 *APOA5* and metabolites (false discovery rate (FDR) < 0.05)

**Supplementary Table 13**. All associations between rs7412 *APOE-APOC1* and metabolites (false discovery rate (FDR) < 0.05)

**Supplementary Table 14**. All associations between rs12916 *HMGCR* and metabolites (false discovery rate (FDR) < 0.05)

**Supplementary Table 15**. All associations between rs17238484 *HMGCR* and metabolites (false discovery rate (FDR) < 0.05)
