# Supplementary material for: Coronary artery disease, genetic risk and the metabolome in young individuals
Source: Wellcome Open Res. 2019 Feb 1;3:114. Originally published 2018 Sep 12. [Version 2] doi: 10.12688/wellcomeopenres.14788.2 (PMC6348437; doi:10.12688/wellcomeopenres.14788.2)
Supplement: Supplementary file 1 [file wellcomeopenres-3-16438-s0000.tgz › 6cde8a13-4546-4748-ab0a-006420f314b5_Supp_figures_revised.docx]

**Supplementary Figures**

**Contents**

Supplementary Figures 1-3………………………………………………………… 2-3

**Supplementary Figure 1.** SNPs associated with one or more metabolites at FDR < 0.05, along with the 2 SNPs within the *HMGCR* region (rs12916, rs17238484).


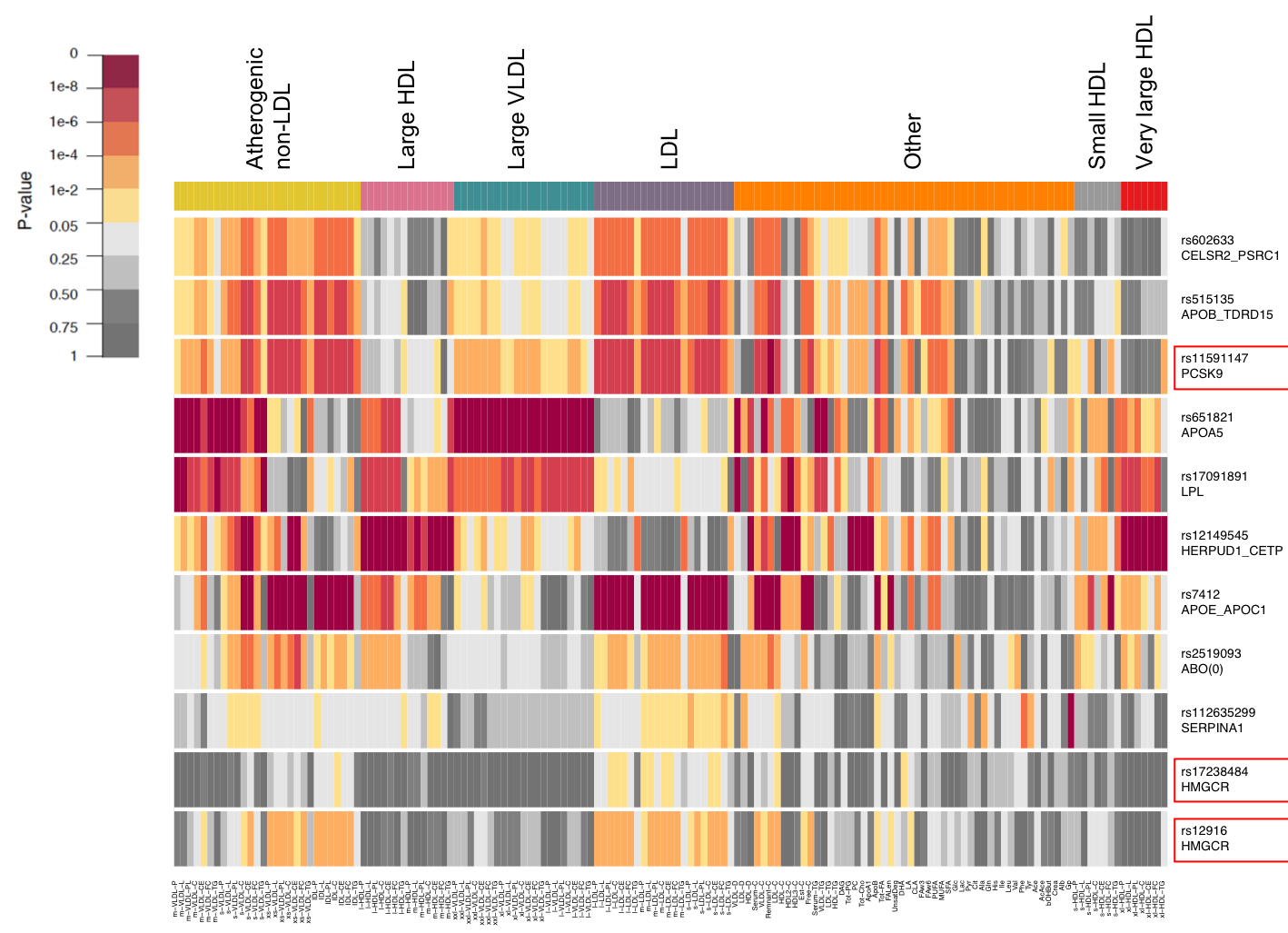

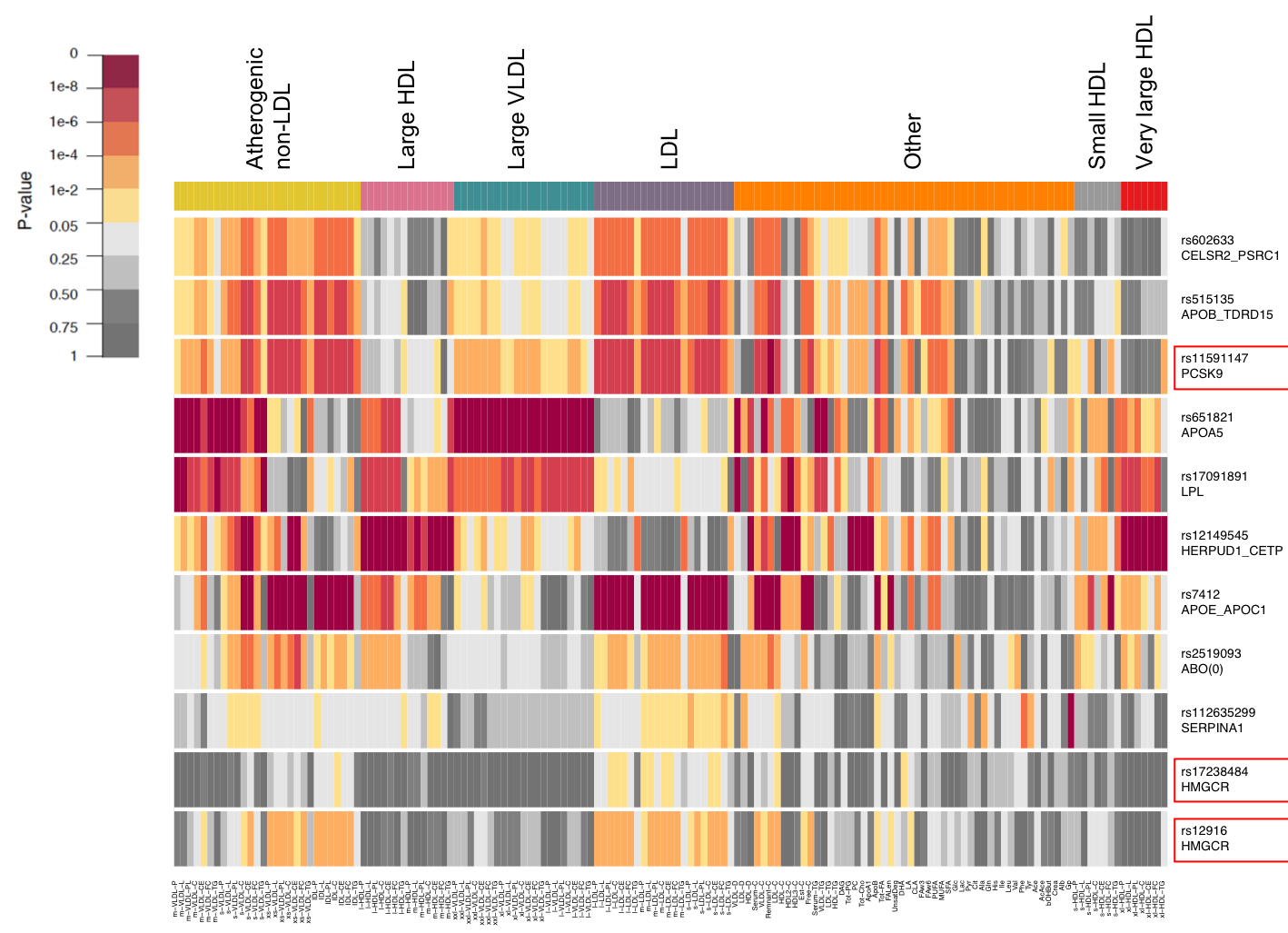


Highlighted are SNPs that are within loci that are targeted by current pharmaceutical agents.

**Supplementary Figure 2.** A forest plot comparing the effect estimates for the association between the CAD genetic risk score and 148 metabolites within each age.

**Supplementary Figure 3.** Comparison of effect estimates (transformed so all estimates are positive) for the association between the CAD-GRS and the lipoprotein sub-groups stratified by age.
