# Supplementary material for: Coronary artery disease, genetic risk and the metabolome in young individuals
Source: Wellcome Open Res. 2019 Feb 1;3:114. Originally published 2018 Sep 12. [Version 2] doi: 10.12688/wellcomeopenres.14788.2 (PMC6348437; doi:10.12688/wellcomeopenres.14788.2)
Supplement: Peer review history

# Peer review: Referee response for version 1

**DOI:** 10.21956/wellcomeopenres.16115.r34125

**Published:** 2018-11-02

**Authors:** Robert Roberts

**Affiliations:** 1 University of Arizona College of Medicine-Phoenix, Phoenix, AZ, USA

The investigators have explored the genetic risk score versus metabolites mainly lipoproteins. This is done in a group of children and adolescents of which there is a scarcity in the literature. The results are worthy of publication and will serve as part of the increasing number of studies, we hope will be performed in this age group in the future. The paper is well done but the conclusions are perhaps overstated. The conclusion that early life intervention for high risk individuals identified by their genetic profile could help prevent onset of the disease is an overstatement for this study. Why? The investigators correlate the genetic risk score with the lipoproteins which is a good segregate for atherosclerosis but is not atherosclerosis per say. The phenotype of atherosclerosis is not assessed. Secondly, there is no intervention in this study to indicate lowering of lipoprotein or the disease. The reviewer would also like to see the raw genetic risk score and how they vary across this population. This should be included in the manuscript not in the supplemental. Secondly, if the data is available, the plasma lipoprotein level should be included. This is of interest for a variety of reasons and is too important to be included in a supplemental. The reviewer also hopes that in the future, at least, the investigators will test the genetic risk score based on non-lipid related genetic risk variants.

I have read this submission. I believe that I have an appropriate level of expertise to confirm that it is of an acceptable scientific standard.

# Response

**Published:** 2019-01-17

**Authors:** Thomas Battram

**Affiliations:** University of Bristol, UK

**Thank you for these positive remarks Dr Roberts. We have addressed your specific comments below.**

 “The conclusion that early life intervention for high risk individuals identified by their genetic profile could help prevent onset of the disease is an overstatement for this study. Why? The investigators correlate the genetic risk score with the lipoproteins which is a good segregate for atherosclerosis but is not atherosclerosis per say. The phenotype of atherosclerosis is not assessed. Secondly, there is no intervention in this study to indicate lowering of lipoprotein or the disease.”

**Thank you for this suggestion. We have now replaced the overstated conclusion with something more reserved. New text:**

**( *CONCLUSION*) “We believe this warrants further research into whether identification of high-risk individuals, identified by their genetic profile, can benefit from increased monitoring and early life intervention, either by pharmaceutical agents or by behavioural changes”**

 “The reviewer would also like to see the raw genetic risk score and how they vary across this population. This should be included in the manuscript not in the supplemental.”

**Thank you for this suggestion. We have now add in the distribution of the genetic risk score amongst individuals within the study.**

**( *RESULTS) *“** **A GRS produced from 146 CAD-associated variants, distribution shown in Figure 1...”**

 “if the data is available, the plasma lipoprotein level should be included”

**We were unsure on the exact suggestion here. The individual level data is only available for those who apply for access with ALSPAC. If associations between the clinical metabolite measures and the GRS is being requested, we have now added a comparison between the two:**

**( *RESULTS*) To assess whether the NMR measurements were representative of what is routinely measured in the clinic, a comparison between clinically measured LDL (a composite of NMR measures) and NMR LDL measures (23 measures) in individuals aged around seven were made. The NMR measures explained 80% of the variance of the composite LDL measure. The effect estimates from the association between all the SNPs and the NMR measures explained 93% of the variance of the effect estimates for the clinically measured LDL and all the SNPs.**

 “The reviewer also hopes that in the future, at least, the investigators will test the genetic risk score based on non-lipid related genetic risk variants”

**Thank you for this suggestion. We did test the association between individual variants of the coronary artery disease genetic risk score and metabolites. This is not looking at “non-lipid related genetic risk variants” specifically, but we would be apprehensive to do this because we’d have to manually pick out which variants are not related to lipids and this could be a matter of contention for many variants as the effect of each variant is not fully understood.**

# Peer review: Referee response for version 1

**DOI:** 10.21956/wellcomeopenres.16115.r33887

**Published:** 2018-10-23

**Authors:** Timothy M. Frayling

**Affiliations:** 1 Genetics of Complex Traits, Royal Devon and Exeter Hospital, University of Exeter Medical School, Exeter, UK

This is a well performed report and I only have minor comments that may help the authors.

 Minor points:

1. I found the penultimate sentence of the results hard to parse.
2. The final sentence of the conclusions gets a little too speculative in my opinion – suggest take out or tone down – e.g. “further research needed to find out if useful, given marker in young children.
3. Introduction – penetrance of alleles can vary across the lifecourse but not sure why the need to suggest gene x environment interactions one source – main source is simple biology – or I guess at best an interaction with age – many monogenic diseases – e.g. Huntington’s don’t manifest until later age. From age 7, after the adiposity peaks and troughs during early growth. FTO does have a relatively stable effect on BMI – see the largest study on longitudinal data from the HUNT study.
4. I am sure you’ve thought of it but if you take the LDL C SNPs, do they have bigger effects in kids compared to old adults? A separate paper and project I imagine, but could be indicative of survival bias in older cohorts, as well as genuine differences in penetrance.
5. Results. Is it surprising that the most strongly associated metabolites in figure 1  - the top 9, are non LDL based? Worth more of a mention in results or discussion (looks like 7 in figure 2)?
6. In the HMGCoR section – is the FDR calculation correct ? 42 / 149 at p<0.05 sounds like an enrichment! Also clarify directions when talking about “inverse associations” – especially in the context of the SNPs.
7. In the discussion you say you found little evidence of association for five SNPs found in adults, and that that could indicate non lipid pathways but could it also be relative lack of power? What were the sample sizes in the GWAS discovery? Presumably much bigger?
8. In the discussion about giving kids statins could mention the specific dangers – myopathy and T2D. 

I have read this submission. I believe that I have an appropriate level of expertise to confirm that it is of an acceptable scientific standard.

# Response

**Published:** 2019-01-17

**Authors:** Thomas Battram

**Affiliations:** University of Bristol, UK

**Thank you for these positive remarks Professor Frayling. We have addressed your specific comments below.**

 “I found the penultimate sentence of the results hard to parse.”

**We’ve now adapted the final sentences of the results to read:**

**( *RESULTS) *“** **The extent of these differences for each metabolite is displayed in Supplementary Figure 2. The effect estimates for associations between the GRS and lipoprotein groups was largely consistent between age groups ( Supplementary Figure 3).”**

 “The final sentence of the conclusions gets a little too speculative in my opinion – suggest take out or tone down – e.g. “further research needed to find out if useful, given marker in young children.”

**Thank you for this suggestion. We have now replaced the overstated conclusion with something more reserved. New text:**

**( *CONCLUSION*) “We believe this warrants further research into whether identification of high-risk individuals, identified by their genetic profile, can benefit from increased monitoring and early life intervention, either by pharmaceutical agents or by behavioural changes”**

 “Introduction – penetrance of alleles can vary across the lifecourse but not sure why the need to suggest gene x environment interactions one source – main source is simple biology – or I guess at best an interaction with age – many monogenic diseases – e.g. Huntington’s don’t manifest until later age. From age 7, after the adiposity peaks and troughs during early growth. FTO does have a relatively stable effect on BMI – see the largest study on longitudinal data from the HUNT study.”

**Thank you for pointing this out. We acknowledge that varying penetrance across the lifecourse could be due to multiple factors so have added that gene x environment interactions is just one source that could provide this variation.**

**( *INTRODUCTION) *Currently, it is often assumed the effect of a genetic variant is fixed across the life course, but whilst the nature of the code itself may be static, the penetrance may be variable, one possible source of this variation comes from gene-environment interactions.**

  “I am sure you’ve thought of it but if you take the LDL C SNPs, do they have bigger effects in kids compared to old adults? A separate paper and project I imagine, but could be indicative of survival bias in older cohorts, as well as genuine differences in penetrance.”

**This is an interesting thought and it would be interesting to address this question for all SNPs measured in the GWAS, but as you mention we think this is beyond the scope of this paper.**

 “Results. Is it surprising that the most strongly associated metabolites in figure 1  - the top 9, are non LDL based? Worth more of a mention in results or discussion (looks like 7 in figure 2)?”

**Thank you for raising this point. Within the top 10, there is only 1 LDL subgroup lipoprotein, but 6 atherogenic non-LDL particles, which we hypothesised to be damaging with regards to cardiovascular health. Thus, we are not too surprised with the result, although did think it deserved a comment as you mentioned:**

**( *DISCUSSION*) Of the nine metabolites that associated most strongly with the GRS, none of them were part of the LDL subgroup, however six were part of the atherogenic non-LDL subgroup previously hypothesised to be dangerous (Supplementary Table 4).**

 “In the HMGCoR section – is the FDR calculation correct ? 42 / 149 at p<0.05 sounds like an enrichment!”

**Thank you for raising this point, I was conservative with the FDR calculation. I adjusted for the fact that I was looking at two SNPs and not one i.e. the FDR calculation was 42/298 at P<0.05. You’re correct in stating that some SNPs would meet the threshold if just one SNP was considered.**

 “Also clarify directions when talking about “inverse associations” – especially in the context of the SNPs.”

**Thank you for this suggestion. This has been corrected throughout the manuscript:**

**( *RESULTS)*** **Mostly, the presence of the effect allele (G and T respectively) associated with a decrease in metabolite levels within the lipoprotein subclasses LDL and atherogenic non-LDL particles.**

**( *DISCUSSION)*** **A previous study by Swerdlow et al. showed that alleles rs17238484-G and rs12916-T ( *HMGCR*) associate with a decrease in LDL-C**

 “In the discussion you say you found little evidence of association for five SNPs found in adults, and that that could indicate non lipid pathways but could it also be relative lack of power? What were the sample sizes in the GWAS discovery? Presumably much bigger?”

**Thank you for raising this point. We have now assessed the difference in effect estimates of our results and the Kettunen et al. GWAS and have added this to the discussion:**

**( *DISCUSSION)*** **In total, in the Kettunen et al. GWAS, the five SNPs associated with 89 metabolites. Of these 89 associations the direction of effect was the same for all but one within children and the 95% confidence intervals overlapped for 80 of the associations. Therefore, the discrepancy between the studies seems to be primarily due to power differences (the GWAS conducted in ~15,000 adults).**

 In the discussion about giving kids statins could mention the specific dangers – myopathy and T2D. 

**Thank you for the suggestion, it has been incorporated into the discussion:**

**( *DISCUSSION)*** **Unfortunately, there may be negative side effects of administering statins to younger individuals, with evidence linking statins to increases in risk of both type 2 diabetes and myopathy.**
